# Supplementary material for: Overexpression of Wild Soybean Expansin Gene GsEXLB14 Enhanced the Tolerance of Transgenic Soybean Hairy Roots to Salt and Drought Stresses
Source: Plants (Basel). 2024 Jun 14;13(12):1656. doi: 10.3390/plants13121656 (PMC11207530; doi:10.3390/plants13121656)
Supplement: Supplementary file 1 [file plants-13-01656-s001.zip › plants-3022279-supplementary.pdf]

Supplementary Table S1

Table S1. The primers used in this study

Name	Sequence (5′-3′)	Length (bp)	Purpose
qRT-GsEXLB14-FW	TACGGTGGCTATAACCTCTT	20	qRT-PCR
qRT-GsEXLB14-RV	ACTCTTGGCAATCTTCCTG	19	
Actin-11-FW	CTTACATTGCCCTTGACTACG	21	Wild soybean *Actin*
Actin-11-RV	AACCTCTGGACATCTGAAACG	21	
GsEXLB14-Clone-FW	CCCTGTGCTTGACACTTTTG	20	Cloning of *GsEXLB14*
GsEXLB14-Clone-RV	CGACTCACCACAAAGGAACA	20	
GsEXLB14-*Sma*I-FW	TCCCCCGGGATGGAACTTAATTTTA	25	Restriction site addition
GsEXLB14-*Sma*I-RV	TCCCCCGGGACCAAGCTGAATTTCG	25	
GsEXLB14-identify-FW	AACACGGGGGACTCTTGAC	19	Identification of expression vector
GsEXLB14-identify-RV	GGGACCAAGCTGAATTTCG	19	
GsEXLB14-transgenosis-FW	GTGTTCAATGCTTTTCAAGATACCC	25	Identification of transgenic soybean hairy roots
GsEXLB14-transgenosis-RV	ACCAAGCTGAATTTCGGAGTCAAA	24	
Actin-FW	AGGATTTGCTGGTGACGATG	20	Soybean *Actin*
Actin-RV	TTTGACCCATCCCAACCAT	19	
